# The Small-Pox

**Published:** 1885-12

**Authors:** 


### The Small-Pox.

In Montreal, the mortality is decreasing. The deaths during the week ending Nov. 4th was 322, against 350 the previous week. The total number of deaths during October was 1,630. Of these, 1,599 were Roman Catholics, and mainly French. It is said that the great mass of people in the French quarter are still unvaccinated. Since the commencement of the epidemic, there have been over 3,000 deaths.

In London, England, for the first time since 1883, a week has passed without the registration of a single death from the disease. Sixteen cases were, however, reported.

One of our exchanges, "The United States Medical Investigator," (homœopathic,) contains an account of an alleged cure of a case of General Cerebral Paralysis. About the only remedies (?) used, were rhus tox. 20th decimal, and rhus tox. 200th decimal. We would be tempted to recommend this treatment of general paralysis of the insane to our readers, were it not for two reasons: First—We doubt the writer's diagnosis of the case, even though "the most prominent physicians of the Old School, in Newark, N. J., agreed as to the nature of the disease;" for there are many cases of subacute mania and melancholia in women, which present symptoms almost identical with those described. The writer, who "frankly admits that he had never treated or seen a case of the kind," should be reminded that many, if not the great majority of, cases of mania and melancholia pass through a stage of dementia, in which, like his case, they become "entirely insensible to cleanliness and decency." Second—Even if his diagnosis is correct, we seriously doubt that his patient is cured. Partial, and sometimes apparently complete, recoveries in these cases is not in our experience at all rare. But these recoveries (?) are only remissions in the progress of the disease.

---

Dr. William B. Carpenter, the eminent English physiologist, author of "A Treatise on General and Comparative Physiology," "The Microscope and its Revelations," etc., died Tuesday, November 10th, in the seventy-third year of his age. Dr. Carpenter died from the effects of burns caused by overturning a spirit lamp while taking a vapor bath.

---

We call attention to the advertisement of the Goodyear Rubber Co., in this issue. Those connected with hospitals, asylums, and other institutions where rubber goods are needed, should not fail to examine the stock of this firm, who manufacture a complete line of syringes, bandages, water bags, and other rubber surgical goods of every kind.

We have received, through the courtesy of Dr. Stephen Y. Howell of this city, a copy of Dr. Friedlander's Manual of Microscopical Technology, which he has translated from the German. It is published by G. P. Putnam's Sons. The translator aims to present to the American student and practitioner more than a literal translation of the German work, and has added thereto much valuable matter which enhances largely its value. It is a pleasure to notice and to commend this effort of our accomplished townsman, and we are sure he has done a valuable service in placing before the profession a trusty guide in a very important department of medical science.

---

We take pleasure in informing our subscribers that Dr. Edward B. Angell will hereafter be our regular correspondent from Rochester, reporting any important papers read before the local societies, and contributing to other departments of the JOURNAL. Dr. Angell received his literary education in the University of Rochester, obtained his medical degree in the University of Pennsylvania, and was, for two years, Clinical Assistant to Dr. S. Weir Mitchell, of Philadelphia.

---

The initial number of the Cleveland *Medical Gazette* is received, and we welcome the new-comer to a place on our table. The editors are Drs. Baker and Kelley. Their modest ambition is "to succeed in editing a live, local medical journal." As our sister city has had for years no local medical journal, there would seem to be a fair field for the enterprise; and if the contents of the succeeding numbers are as good as this one, the *Gazette* will deserve success.

---

The office of the JOURNAL has been removed to more convenient quarters at No. 369 Pearl street. Our correspondents will please make a note of the change of address.

We call attention to advertisement of Messrs. Denny & Field, Druggists. These gentlemen are both graduates of pharmacy and well qualified for their important duties. We have more than once had occasion to commend the excellent quality of drugs supplied by them, as well as to note the moderate prices which our patients have commented upon. They are deserving of success.

